# Post-translational Modifications of the p53 Protein and the Impact in Alzheimer’s Disease: A Review of the Literature

**DOI:** 10.3389/fnagi.2022.835288

**Published:** 2022-04-28

**Authors:** James S. Clark, Rakez Kayed, Giulia Abate, Daniela Uberti, Paul Kinnon, Simona Piccirella

**Affiliations:** ^1^Diadem srl, Brescia, Italy; ^2^Mitchell Center for Neurodegenerative Diseases, The University of Texas Medical Branch, Galveston, TX, United States; ^3^Department of Neurology, Neuroscience and Cell Biology, The University of Texas Medical Branch, Galveston, TX, United States; ^4^Division of Pharmacology, Department of Molecular and Translational Medicine, University of Brescia, Brescia, Italy; ^5^Department of Molecular and Translational Medicine, University of Brescia, Brescia, Italy

**Keywords:** p53, Alzheimer’s disease (AD), post translational modification (PTM), review, TP53

## Abstract

Our understanding of Alzheimer’s disease (AD) pathogenesis has developed with several hypotheses over the last 40 years, including the Amyloid and Tau hypotheses. More recently, the p53 protein, well-known as a genome guardian, has gained attention for its potential role in the early evolution of AD. This is due to the central involvement of p53’s in the control of oxidative stress and potential involvement in the Amyloid and Tau pathways. p53 is commonly regulated by post-translational modifications (PTMs), which affect its conformation, increasing its capacity to adopt multiple structural and functional states, including those that can affect brain processes, thus contributing to AD development. The following review will explore the impact of p53 PTMs on its function and consequential involvement in AD pathogenesis. The greater understanding of the role of p53 in the pathogenesis of AD could result in more targeted therapies benefiting the many patients of this debilitating disease.

## Introduction

### Pathology of Alzheimer’s Disease

Alzheimer’s disease (AD) is a pernicious condition of the aging population that leads to progressive degeneration of brain function, whose characteristic anatomopathological hallmarks, firstly described by Alois Alzheimer, remain the senile plaques and neurofibrillary tangles which consist of beta-amyloid peptides and hyperphosphorylated Tau proteins (Querfurth and LaFerla, 2010). The formation of brain plaques begins up to 20 years before any signs of clinical symptoms. To date, our understanding of AD pathology is still incomplete, with several different models proposed (Jack and Holtzman, 2013; Fagan et al., 2014).

The amyloid hypothesis of AD pathology suggests that the increased presence of amyloid-beta (Aβ) is the triggering factor, which then links directly to Tau protein hyper-phosphorylation, synapse loss, and cell death (Gao et al., 2018).

However, the classical amyloid hypothesis has been questioned (Ricciarelli and Fedele, 2017) and other hypotheses have now been postulated to describe AD pathology and are discussed in the following sections.

GSK-3β is a critical cell cycle regulation factor and has an important function in neurons for Tau regulation. Increased GSK-3β activity has been shown to be one of the first events in AD pathology, leading to increased Aβ production and Tau hyper-phosphorylation (Braak and del Tredici, 2004). Moreover, the ε4 isoform of Apolipoprotein E (Apo E4), which inhibits proteolytic degradation of Aβ, has also been implicated as a significant genetic risk factor for the sporadic AD (Mulder et al., 2014; Johnson, 2020). In addition, glutamatergic and cholinergic dysfunction (Howes and Houghton, 2012), oxidative stress (Cheignon et al., 2018), prion proteins (Westergard et al., 2007), α-synuclein (Twohig and Nielsen, 2019), TDP-42 (Tomé et al., 2020), and inflammation (Latta et al., 2015) are all implicated in AD pathology.

Mitochondrial dysfunction has also been implicated in AD pathology, Oxidative stress, damage by free radicals in parallel with changes in the expression of superoxide dismutase (SOD) and catalase, both important anti-oxidant enzymes, have been shown in tissue from AD patients (Marcus et al., 1998; Omar et al., 1999; Padurariu et al., 2010).

Interestingly, In the AD brain, fatty acid oxidation has been linked to Tau pathology, and p38 MAPK has been linked as a potential candidate gene within the relevant MAP Kinase pathway (Zhu et al., 2000). Further studies have implicated oxidative stress in Aβ induced neurotoxicity (Mattson, 2007) and cellular (Behl et al., 1994) and rodent (Li et al., 2004; Nishida et al., 2006) models showed increased oxidative stress is linked to Aβ deposits. Accumulation of transition metals along with Aβ and Tau protein have all been implicated in the loss of redox balance, and oxidative stress is a well-recognized feature of AD brains (Praticò, 2008). The metal theory of AD (Deibel et al., 1996; Bush, 2013) proposes that homeostasis of transition metals is severely altered in AD, with extracellular pooling of metals such as Zn and Cu in amyloid, and intraneuronal accumulation of Fe within the AD brain. Abnormal levels of metal ions, Zn, Cu, and Fe were shown in AD brains (Deibel et al., 1996). Also, the presence of transition metals has been highlighted in amyloid deposits and transgenic murine models (Lovell et al., 1998; Zhang et al., 2006).

The development of clinical symptoms of AD requires many decades for pathological changes to develop (McDade et al., 2018). However, during the early stages of the disease, the body’s cellular defense mechanisms, including the DNA damage response, will activate and potentially stop the progression of AD (Jackson and Bartek, 2009).

As seen in cancer, cellular response mechanisms involve multiple pathways. The role of one of these genes, TP53, is now being revealed as central to the cellular response to cancer and other diseases, including obesity and aging-associated neurodegenerative disorders (Labuschagne et al., 2018). Given the implication of different mechanisms of cellular stress in AD pathology, understanding the role and regulation of TP53 and its protein product p53 in AD could unlock therapies to treat the disease in its early stages or facilitate AD diagnosis before the clinical manifestation of the disease.

### p53 Conformational Changes: Causes and Consequences in Alzheimer’s Disease

Ever since the discovery of p53 (Lane and Crawford, 1979; Linzer and Levine, 1979) it has been accepted as a central tumor suppressor that has also been shown to be involved in diseases other than cancer, acting as a critical gatekeeper for cellular stress. p53 is a homo-tetrameric, multidomain transcriptional factor and, by binding of the p53-responsive elements found at target genes’ promoters, p53 can in turn activate multiple pathways and genes (Liu et al., 2019).

Tumorigenesis of p53 has been shown in numerous mouse experiments where p53 loss-of-function predisposes cells to permanent damage and tumor transformation (Donehower et al., 1992; Olive et al., 2004). Tumorigenesis has also been shown in p53 wild-type patients, whose cellular p53 pathways are not functional, due to several causes (Yue et al., 2017). It has also been demonstrated that p53 functions as a central hub to deal with cellular oxidative stress. In addition to DNA damage, p53 can also show a response to many upstream stress signals which can include oncogene activation, erosion of telomeres, and hypoxia (Horn and Vousden, 2007). When activated, p53 is a regulator of multiple cellular processes including cell cycle arrest, DNA repair, apoptosis, ferroptosis, senescence, that promote cell survival, or minimize cellular malignant transformation.

p53 has been shown to have further roles including the control of cell metabolism, pluripotency, epigenetic control, and aging (Kastenhuber and Lowe, 2017). As p53 has many roles in cellular maintenance, any alteration of functional activity will greatly affect its downstream processes (Vousden and Lane, 2007).

The role of p53 is now realized to be much greater than simply dealing with DNA damage. Thus, p53 should now be regarded not only as a “genome guardian” but as a “stress guardian.”

There are several cellular mechanisms that control p53 function, and post-translational modifications (PTMs) are the most common and effective type of regulation (Chen et al., 2020).

### p53 Post-translational Modifications of the Linear Sequence and the Related Biochemical Reactions

**FIGURE 1:**
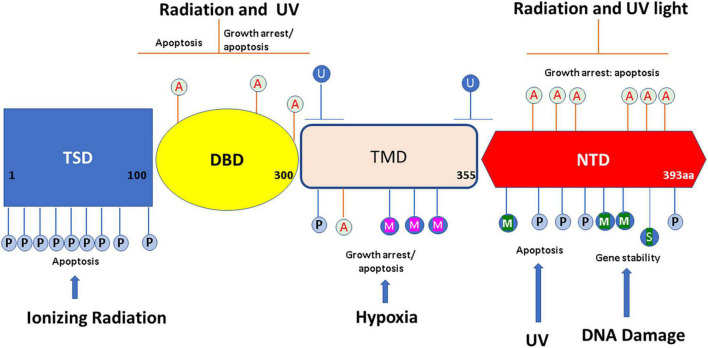
Overview of p53 post-translational modifications (PTMs). The major sites for p53 modifications (β-hydroxybutyrylation, SUMOylation, methylation, hydroxylation, ubiquitination, neddylation, acetylation, *O*-GlcNAcylation, ADP-ribosylation, and phosphorylation) are shown in the figure, with different colors to denote different modifications. TSD, trans activation domain; DBD, DNA binding domain; TMD, tetramerization domain; NTD, N terminal domain. This figure has been modified from Chen et al. (2020).

p53 protein’s modular structure is optimized for PTMs including SUMOylation, neddylation, phosphorylation, acetylation, methylation, ubiquitination, hydroxylation, *O*-GlcNAcylation, ADP-ribosylation, and β-hydroxybutyrylation (Chen et al., 2020; Figure 1).

#### Phosphorylation

p53 Phosphorylation occurs mainly at serine and threonine residues at both C and N-termini. It has been shown that phosphorylation is critical at Ser15 to allow MDM2 dissociation with the p53 protein, with the result of stabilizing the p53 complex (Shieh et al., 1997). AMP-activated protein kinase (AMPK) pathway can phosphorylate Ser15 and mediates a G1/S cell cycle block (Jones et al., 2005). p53 phosphorylation at position 392 can be induced by DNA damage and has been shown to activate the DNA binding capacity of p53. Overall, phosphorylation of p53 can alter the formation and conformation of p53 tetramers, which in turn, can alter their activity (Chène, 2001).

#### Acetylation

Acetylation of eight lysine residues of p53 (K120, K164, K370, K372, K373, K381, K382, and K386) are induced by cytotoxic stimuli, which results in p53 activation and stabilization (Barlev et al., 2001; Brooks and Gu, 2011a). p53 acetylation is important for the control of the cell cycle, especially the G2 cell cycle phase (Imbriano et al., 2005). Acetylation of the lysine residues present at the C-terminal inhibits ubiquitination by MDM2 on the same residue. This stabilizes the tetramer and improves its DNA binding ability, allowing transcriptional activators to be recruited (Itahana et al., 2009). p53 transactivation is reliant on a variety of promoters, with acetylation dependent on the cellular environment.

#### Methylation

Methylation of lysine and arginine residues is an important control mechanism of p53 function as it’s a reversible process (Jansson et al., 2008). Interestingly, nuclear lysine methylation occurring after damage to DNA enhances p53 chromatin binding; increasing p53 recruitment in p21 regulatory regions; enhancing p21 activation along with other downstream genes (Chuikov et al., 2004). p53 activity is also modulated by arginine methylation and this mechanism regulates the p53 response. Like lysine methylation, arginine methylation of p53 is also strongly linked to DNA damage (Jansson et al., 2008). Methylation by PRMT5 is also known to influence the activity of p53 oligomerization (Jansson et al., 2008).

#### Ubiquitination

MDM2 ubiquitination is critical for maintaining p53 levels in the cell. This has been postulated to occur by polyubiquitination of p53 leading to proteasome pathway mediated protein degradation, which then inhibits p53-mediated transactivation (Haupt et al., 1997; Brooks and Gu, 2011b). The major MDM2 ubiquitination sites of p53 have been shown to be located at the carboxy end (Rodriguez et al., 2000). Interestingly, expression of MDM2 is also regulated by p53, creating a negative feedback loop. Thus, increasing the cellular p53 level will induce MDM2 expression, and subsequently lead to a decrease in p53 expression and loss of activity (Wu et al., 1993). Additionally, work by Lee et al. (2008) showed that ubiquitination inhibits p53 binding leading to apoptosis and finally cell cycle arrest.

Different types of p53 ubiquitination are known to cause changes in p53 function as well as stabilization, by regulating its cytosolic location and nuclear export. p53 ubiquitination is also implicated in the disruption of its binding to promoters of target genes, similar to the action of transcription factors in the nucleus. This results in halting the cell cycle and triggering apoptosis (Lee and Gu, 2009). In unstressed cells, levels of p53 protein are maintained at low levels due to ubiquitination of MDM2 and MDM4, which target p53 for proteasomal degradation. TP53 PTMs modulate TP53 stability as well as its interactions with DNA, chromatin, and many cofactors that influence TP53-mediated transcription or repression of target genes. TP53 is also known to transcriptionally activate a large number of genes by binding to response elements that are found proximal to promoters/enhancers (Riley et al., 2008).

#### Other Modifications

These include neddylation and SUMOylation (Xirodimas et al., 2004; Abida et al., 2007; Wu and Cheng, 2009).

These modifications all have several common features: (i) Multiple sites: on many different amino acids. (ii) Multiple functions: that are site-specific. (iii) Reversibility: such that there are functionally modifying and de-modifying mechanisms. (iv) Cross talk: modifications at certain sites can affect modifications at other sites (Liu et al., 2019).

#### Nitrosylation

Aerobic metabolism has several by products including ROS and reactive nitrogen species (RNS). RNS can initiate signaling that is transduced through redox-based PTMs of proteins. It is known that redox signaling is a critical process in cell physiology and is an important regulator of redox-sensitive proteins (Metodiewa and Kośka, 1999). The brain is a major producer of RNS which produce two main types of protein modifications, nitrosylation of thiol groups (S-nitrosylation), and nitration of tyrosine residues (nitroTyr). S-Nitrosylation is a specific and reversible modulation that occurs on a physiologically relevant timescale. Target depending, this results in either activation or inhibition of cellular pathways (Janssen-Heininger et al., 2008). It is proposed that S-nitrosylation of Cys residues is the redox-based post-translational mechanism equivalent to protein phosphorylation (Hess et al., 2005). In contrast, abnormal S-nitrosylation could be an important factor in brain aging and the development of neurodegenerative disease (Finelli, 2020).

#### Tyrosine Nitration and Alzheimer’s Disease Pathogenesis

Data suggests a strong link between protein Tyr nitration and the pathogenesis of AD. For example, Buizza et al. (2013) demonstrated an elevated expression of nitrated p53 in an *in vitro* model of amyloid precursor protein (APP) overexpression-induced Aβ load. Concomitantly, they observed an associated increase in the expression of unfolded p53, and neuronal dysfunction. Restoring the native conformational state of p53 with Zinc chloride prevented neuronal dysfunction, supporting the role of p53 in maintaining neuronal functionality (Buizza et al., 2013). In further studies the unfolded p53 variant was identified in AD patients (Uberti et al., 2006; Buizza et al., 2012; Abate et al., 2020a). The review of Abate et al. (2020b) describes the potential role of p53 in AD pathogenesis and diagnosis. This review will discuss the functional role of p53, in particular post translational modifications and AD.

### p53 Function and the Link to Alzheimer’s Disease

**FIGURE 2:**
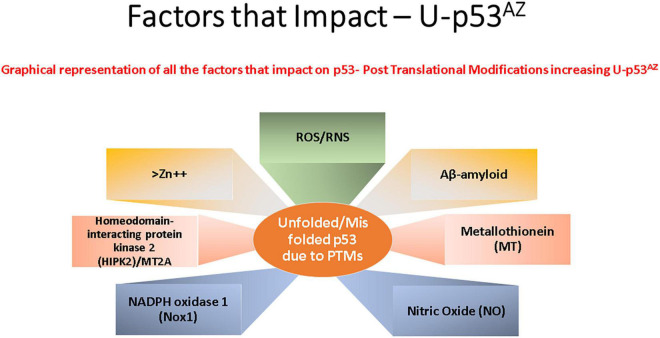
Key factors that affect the generation of the conformational change of p53 to an unfolded conformation in the current model understanding of Alzheimer’s disease (AD). Each box refers to a significant cellular factor linked to PTMs of the p53 protein.

p53 protein function has been shown to be closely associated with many cell stress control mechanisms and is proposed to be intrinsically involved in defense against neuronal degeneration. These mechanisms include maintenance of redox control, redox homeostasis, and inflammation, along with regulation of the neuronal cell cycle and control of Aβ peptides (Lanni et al., 2007). Thus, deregulation of a central protein such as p53 could significantly contribute to neuronal dysfunction and play a critical role in neurodegeneration. Figure 2 summarizes the main factors that can impact p53 conformation and highlights within the current models of AD pathogenesis the main areas implicated and referenced in detail in the sections following.

#### Oxidative Stress

It is well established that p53 is involved in redox homeostasis and that loss of its biological activity, caused by changes in its conformational structure, leads to exacerbation of oxidative stress (Zhao and Zhao, 2013). Growing evidence proposes oxidative stress to be a critical factor in both AD initiation and progression with a strong relationship to Aβ and Tau-induced neurotoxicity forming feedback loops to accelerate AD progression (Mocchegiani et al., 2004; Puca et al., 2008, 2010a; Lanni et al., 2010a; Zhao and Zhao, 2013).

The cysteine rich metallothionein protein has been implicated in the cellular detoxification of inorganic species, accomplished by sequestering metal ions present in increased concentrations. Specifically, this was shown in the hippocampus of old rats where metallothionein isoforms I + II and III and interleukin-6 found in the hippocampus of old rats, where increments in their expression could potentially lead to neurodegeneration (Mocchegiani et al., 2004). NADPH Oxidase 1 (Nox1) has also been postulated to have a role in p53 deacetylation, along with the suppression of transcriptional activity and apoptosis (Puca et al., 2010b). Chronic Cerebral Hypoperfusion (CCH) has revealed Nox1 is activated in the hippocampus, causing oxidative stress, hippocampal neuronal death, and then cognitive impairment. Implying Nox1-mediated oxidative stress has a critical role in neuronal cell death and cognitive dysfunction in dementia (Choi et al., 2014).

#### Amyloid Accumulation

Amyloid plaque accumulation in the AD brain has also been linked to p53 (LaFerla et al., 1996; Lapresa et al., 2019; Abate et al., 2020a; Farmer et al., 2020). This is not surprising due to the many functions of p53 in its role as a “cellular guardian.” Any Deregulation will affect many signaling pathways implicated in AD, with p53 at the crossroads of a complex network of stress response pathways (Stanga et al., 2010). The Aβ-induced depletion of Homeodomain Interacting Protein Kinase 2 (HIPK2) has been demonstrated to be involved in p53 conformational changes toward an unfolded state, thus contributing to cellular dysfunction (Puca et al., 2008, 2010b; Lanni et al., 2010a). In detail, sublethal amounts of Aβ inhibit the Zyxin/HIPK2 signaling axis, upregulating MT2A resulting in chelated zinc atoms causing a change in p53 conformation (Abate et al., 2020a). HIPK2 modulation leads to the up-regulation of MT2A, which chelates Zn ions leading to p53 conformational change (Lanni et al., 2010a,^2013^). In the review of Méplan et al. (2000) further support for the role of Zn ions in this process is evidenced by Zn^2+^ redox signaling and transition metals being linked with p53 pathway regulation (Méplan et al., 2000), where wt-p53 activity was restored in HIPK2-depleted cells by altering metallothionein and zinc levels (Puca et al., 2009).

#### Inflammation

An inflammatory environment has been linked to, specifically an immune infiltrate localized to amyloid plaques within the AD brain to AD pathology (Kinney et al., 2018). The presence of a highly active immune response in the AD brain, consisting of macrophages and other immune cells, has been implicated in amyloid and Tau pathology, with microglia-related signaling mechanisms hypothesized to be involved in AD. However, the exact mechanisms involved still need to be elucidated (Kinney et al., 2018).

#### Cell Cycle Deregulation

Cell cycle deregulation has been implicated in AD as a greater number of dividing cells have been observed in AD brains vs. controls (Zhou and Jia, 2010). The cell cycle hypothesis may explain the slow rate of atrophy during the progression of AD (Zhou and Jia, 2010).

**FIGURE 3:**
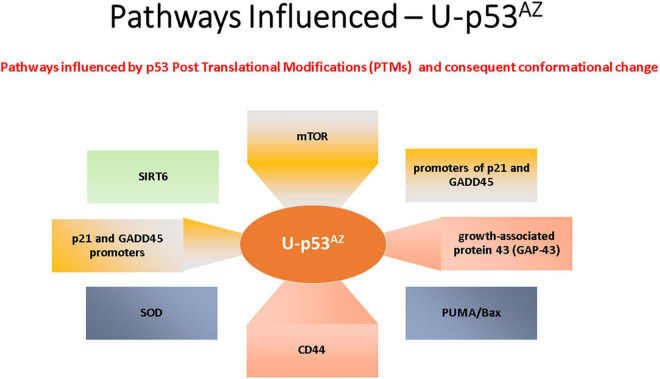
Summary of the main signaling pathways that are affected by the PTM-induced conformational changes causing unfolded p53.

The areas previously discussed are all regulated by p53, and deregulation with a subsequent loss of function due to a PTM-related conformational change to unfolded p53 could link p53 to the pathogenesis of AD. The impact of the unfolded conformational variant of p53 is summarized in Figure 3, where the implicated AD pathways are directly linked to the specific factors related to the conformational change in p53.

In addition, the functional p53 protein is critical to ensuring that aging cells are suitably regulated. With most of the research over the last 40 years completed on p53 and cancer (Feng et al., 2011; Wu and Prives, 2017) and more recently in other disease areas, murine studies have been extensively used to study aging with a number focusing on isoforms of p53. Findings indicate that over-expression of terminally truncated p53 (as p44) protein is potentially linked to aging and aggregation of Tau in NFT’s (Pehar et al., 2010, 2014). These aged mice also had synaptic deficits and a similar decline in cognitive function to AD, and interestingly a significant increase in Tau phosphorylation suggesting a Tau/p53 link in AD pathogenesis (Pehar et al., 2010, 2014). Within the human AD brain, Turnquist et al. (2016) have implicated changes in expression of p53-beta and delta 133 p53 isoforms in the pathology of AD brains compared to controls (Turnquist et al., 2016).

### p53 Signaling Pathways in the Pathogenesis of Alzheimer’s Disease

As discussed in the previous section, the main regulation of p53 is by protein-protein interaction, and many PTMs can regulate p53 activity and could be a critical point of dysregulation during the development of AD (Chen et al., 2020).

During cellular stress, the p53 response can recruit a number of key signaling proteins, including cell cycle checkpoint inhibitors and others (Shieh et al., 1997; Budanov, 2014).

#### Cellular Damage

The normal cellular defense activity of p53 is an action to repair the cellular damage or to lead the cell to apoptosis; these are critical processes during cellular aging (Budanov, 2014). Interestingly, the expression of ataxia-telangiectasia mutated (ATM) was shown to be increased in a range of mild to moderate to severe AD patients compared to controls and correlated with plaque density and Braak stages (Katsel et al., 2013). These data strongly indicate an active p53 mediated DNA damage response is being carried out in AD.

#### Oxidative Stress

The physiological function of p53 is to respond to oxidative stress, as shown by the activation of antioxidant genes and their associated signaling pathways. These pathways include Manganese Super Oxide Dismutase (MnSOD) and the TP53 induced glycolysis and apoptosis regulator (TIGAR) (Sablina et al., 2005; Budanov, 2014). An impaired antioxidant response, with low MnSOD activity was shown in an AD mouse model (Sompol et al., 2008). However, there are several conflicting studies showing expression of TIGAR is inversely correlated with the severity of AD, although in mild dementia, downregulation of TIGAR was noted in the superior temporal cortex (Sablina et al., 2005; Katsel et al., 2013).

#### Aβ Generation

*In vitro* experiments on BACE, the rate-limiting enzyme in the generation of Aβ, showed its transcription is repressed by p53, thus affecting potential Aβ accumulation. During oxidative damage exposure, p53 expression is upregulated. This leads to a decrease in the level of BACE1 expression and potentially contributes to AD pathogenesis (Singh and Pati, 2015).

#### mTOR Signaling

The mTOR signaling pathway has been implicated many times in p53 activity, and interestingly, this pathway has been shown to be activated in early AD before dementia (Feng et al., 2005; Li et al., 2005; Yates et al., 2013; Sidorova-Darmos et al., 2018). An AD transgenic mice study has indicated that a build-up of Aβ increased the function of the mTOR signaling pathway resulting in a negative feedback loop inhibiting autophagy and thus the clearance of Aβ peptides. This evidence suggests that activation of the mTOR pathway may be caused by dysregulated p53 but requires further studies to confirm this hypothesis.

#### Sirtuin Pathway

A member of the sirtuin family (SIRT1), is known to regulate cellular metabolic activity through the process of deacetylation/acetylation. Sirtuins are NAD+-reliant deacetylases. Sirtuin deficiency disrupts mitophagy in numerous diseases, including AD (Sidorova-Darmos et al., 2018).

A recent publication has shown another potential p53 regulated pathway involved in AD development involving sirtuin 6 (SIRT6). The SIRT6 pathway promotes cell longevity by controlling several different processes including energy metabolism, genome integrity and inflammation, along with DNA repair. Interestingly a reduction in the level of the SIRT6 protein has been observed in human AD brain samples (Jung et al., 2016). The control mechanism was investigated in a neuronal cell line, where Aβ 42 post-transcriptionally reduced p53 protein levels resulting in decreased binding of p53 to the SIRT6 promoter, downregulating the p53 gene (Jung et al., 2016).

Upregulation of p53 protein by Nutlin-3 avoids SIRT6 decline, and DNA impairment induced by Aβ (Jung et al., 2016).

Sirtuin family is both a stimulatory and inhibitory factor linked to p53 and, in postnatal cortical neurons, the p53 related apoptosis required PUMA transcriptional activation. PUMA, along with the Bax-mediated permeabilization of the mitochondrial outer membrane, induces cytochrome C release, caspase activation, triggering cell death. SIRT1 inhibits the apoptosis pathway by deacetylation of p53 in several senescence models (Talebi et al., 2021).

#### Cyclin Dependent Kinase

Interestingly, a recent study showed the exposure of neurons to fragments 25–35 of Aβ peptide activates Cdk5, which promotes p53 phosphorylation and stabilization (Lapresa et al., 2019). Mitochondrial dysfunction and neuronal apoptosis were prevented when Cdk5 and p53 functions were inhibited. Thus, Cdk5 is activated by fragments 25–35 of Aβ inducing p53 phosphorylation and stabilization, and this could be an attractive therapeutic target against an Aβ induced neurodegeneration feedback loop (Lapresa et al., 2019).

#### p53 Conformational Change

The first evidence of a conformational change of p53 in AD was shown by Uberti et al., 2002. They showed that an AD conformational change in p53 occurs in skin fibroblasts isolated from AD patients. Upon the exposure of these skin fibroblasts to hydrogen peroxide (H_2_O_2_), there is no activation of p53 dependent cell cycle regulators p21, GADD45, and BAX1 genes (Uberti et al., 2006). This impairment resulted in an accelerated re-entry and diminished H_2_O_2_-related apoptosis when compared to control fibroblasts. However, this mechanism was not initially shown in neurons but clearly showed DNA damage repair mechanism impairment in peripheral cells. Loss of p53 activity was due to a change in the tertiary structure of the protein as demonstrated by the use of anti-p53 conformational specific antibodies. It was later confirmed that the p53 gene was not mutated, and only the protein conformation had been changed (Cordani et al., 2016). The authors proposed this effect was AD specific as *in vitro* models expressing the APP-751 protein exposed to Aβ 1–40 and Aβ 1–42, as well as fibroblasts derived from healthy non-demented subjects, also exposed to Aβ 1–40 and Aβ 1–42, had the same conformational changes. These changes were specific to AD when compared to other dementias and cancer. The proposed mechanism was linked to the Zyxin pathway with Aβ peptides being implicated in deregulating the Zyxin protein leading to proteasomal degradation and downregulation of HIPK2 and the up-regulation of MT2A, which chelates Zn ions leading to p53 conformational change. This is a reversible process with the addition of Zn ions increasing the cellular sensitivity to both cytotoxic damage and the level of growth-associated protein 43 (GAP-43) (Lanni et al., 2010a; Buizza et al., 2013). The conformationally changed variant, an unfolded version of p53, increases APP metabolism and associated Aβ load. Increased expression of unfolded p53 results from the loss of p53 proapoptotic activity in parallel to a decrease in mRNA and protein levels of GAP-43 (Buizza et al., 2013). As a control, treating HEK cells with an Aβ sequestering antibody resulted in the partial prevention of the p53 conformational change indicating a direct link to Aβ (Uberti et al., 2007). Using immortalized lymphocytes from AD patients induced nitration of specific tyrosine residues conformational change of p53 (Buizza et al., 2012). Studies utilizing lymphocytes from AD patients showed an increase in CD44 when increased p53 is also observed, which may also link the lymphocytic control pathways. The evidence supports the hypothesis that the conformational change of p53 could be a significant contributor to the dysregulation of the mTOR AD signaling pathway previously discussed in this review.

The main AD signaling pathways implicated with p53 conformational change are summarized in Figure 3 and are referenced in detail in this section.

#### Relationship Between Tau, Aβ, and p53

**FIGURE 4:**
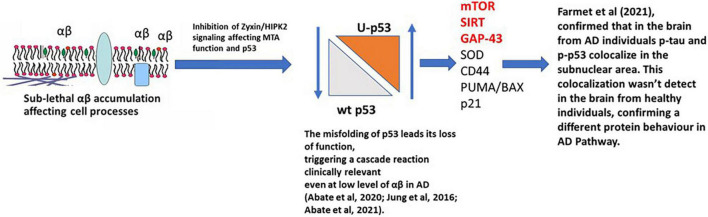
p53 hypothesis in AD. Sub-lethal accumulation of Aβ affects cellular pathways leading to the misfolding of p53 and its loss-of-function, triggering a clinically relevant cascade reaction. In the AD brain, phosphorylated p53 (p-p53) and p53 aggregates are overexpressed compared to the healthy individuals (controls), confirming a different protein behavior than the WT isoform. mTOR, SIRT, and GAP-43 highlighted in red are postulated to be the major pathways involved.

The relationship between Tau, Aβ, and p53 has been explored in many studies and has been considered as a potential therapeutic target for AD (Jazvinšćak Jembrek et al., 2018). The recent manuscript from Sola et al. (2020) focused on the effect of Tau on p53 function during the DNA damage response, studying acute DNA damage in Neuroblastoma cells after Tau depletion. These conditions altered the stability and activity of p53 resulting in reduced cell death, confirming the interaction with p53 and its E3 ubiquitin ligase MDM2 (Sola et al., 2020). Farmer et al. (2020) further investigated the potential role of Tau and p53 in AD to an impaired DNA damage response. Farmer et al. (2020) demonstrated that p53 forms oligomers and fibrils in human AD brain but not in control brains and that the p53 protein interacts with Tau oligomers in AD. p53 oligomers also colocalize, potentially seeding endogenous p53 in primary neurons in the presence of DNA damage, and phosphorylated p53 was shown to be blocked outside the nucleus. p53-mediated DNA damage responders were also shown to be decreased in the AD brains, with control brains showing a normal DNA damage response mechanism. This could indicate that in AD a loss of p53’s nuclear function may be due to p53 aggregation and tau oligomer interactions. A linear p53 hypothesis is proposed in Figure 4.

When Aβ is present at nanomolar levels, through the HIPK2 inhibition, it induces metallothionein 2A expression and, with Zn^2+^-chelating activity, metal ions are sequestered from the p53 DNA binding domain, inducing conformational changes in p53, inhibiting its activity. This directly links to Farmer et al. (2020), where the interaction of p-Tau, p-p53, and the localization of p-p53 outside the nucleus, were observed.

A link between the p53 Conformational Variant and Aβ by HIPK2–p53 signaling was confirmed by an *in vitro* study (Jazvinšćak Jembrek et al., 2018). Furthermore, a study using a pro-oxidant environment activated p53 intracellular pathways, affecting tertiary protein structure, inducing conformational changes and the loss of its activity (Abate et al., 2020b).

p53 regulates a heterogeneous variety of biological functions, which includes neuronal activities including outgrowth and protection of connectivity, and redox homeostasis (Li et al., 2019), these findings reinforce the hypothesis that loss of function of p53 due to its conformational change in the early stages of AD may contribute to several associated pathologies including synapse dysfunction, inflammation, and oxidative stress. Therefore, the p53 conformational variant could be a biomarker of early AD pathological events. These will include the accumulation of Aβ, redox deregulation, and immune activation leading to oxidative stress and chronic inflammation. The link of p53 and its conformational variant, caused by PTMs and linked to Tau and Aβ, is summarized in Figure 4.

### the Conformational Variant of p53 Detected in Alzheimer’s Disease

The importance of the unfolded conformational variant of p53 caused by PTMs has been investigated in several studies comparing AD patients to controls, and a significantly higher expression of the conformational variant of p53 was detected in peripheral blood mononuclear cells (PBMCs) from AD patients (Uberti et al., 2008; Lanni et al., 2010b; Stanga et al., 2012). Interestingly, unfolded p53 was independent of apolipoprotein E epsilon 4 (APOE ε4) status (Abate et al., 2020a). p53 levels were increased in PBMCs from preclinical patients with mild cognitive impairment (MCI) when compared to controls. This led to a study indicating increased levels of unfolded p53 protein were highly predictive of the conversion from amnestic MCI to AD dementia (Uberti et al., 2008; Lanni, et al., 2010a; Stanga et al., 2012).

As previously discussed, the expression of the unfolded conformational variant of p53 was investigated in other dementias, and other brain disorders such as Parkinson’s disease, where the data indicated that p53 is differentially expressed when compared to AD patients, thus suggesting specificity for AD (Uberti et al., 2008).

To further investigate the relationship of unfolded p53 isoforms in AD, Memo and Uberti (2018) developed a monoclonal antibody (mAb) 2D3A8 to be highly specific for the p53 conformational variant and targeted to potential nitrosylation sites. The 2D3A8 antibody was characterized and shown to recognize a linear epitope between the p53 DNA binding domain and the conjunction region with the tetramerization domain. The authors proposed this epitope becomes exposed due to redox PTMs due to the pro-oxidant environment in AD.

The recent study by Abate et al. (2021) further evaluated the presence of the conformational variant of p53 using the mAb 2D3A8. Three hundred seventy-five plasma samples from two longitudinal AD studies were used in conjunction with a machine learning approach to create an algorithm to predict AD likelihood risk. The concentration of p53 conformational variant detected by 2D3A8 and quantified by an in-house plasma ELISA method, Mini-Mental State Examination (MMSE), and levels of APOE ε4 were considered. Applying machine learning, the algorithm identified an AD likelihood risk that showed a robust 86.67% agreement with clinical diagnosis. A highly significant area under the curve (AUC = 0.92) was found for amnestic Mild Cognitive Impairment (aMCI) patients who will develop AD, suggesting 2D3A8-postive p53 as a prognostic biomarker for AD.

The conformational variant of p53, detected by 2D3A8 and then quantified by mass spectroscopy (identified as U-p53^AZ^), was further validated as a potential prognostic biomarker in AD in a set of plasma samples from the longitudinal and retrospective biobank, AIBL (Piccirella et al., 2021). In detail, 482 individuals (515 total samples) up to 144 months after baseline and at different stages of cognitive decline due to AD were analyzed. The U-p53^AZ^ was shown to have a high prognostic value, predicting the progression to AD from preclinical or prodromal AD with a significant AUC > 0.90, showing prognostic validity more than 6 years prior to signs of clinical symptoms. Additionally, the prognostic performance of this conformational variant of p53 was shown to be higher than other main risk factors alone or in combination with amyloid status. U-p53^AZ^ was also shown to have high diagnostic performance to segregate cognitively normal individuals from those with AD (AUC values > 0.90).

## Conclusion

The exact pathology of AD is controversial and remains to be fully elucidated. The validity of the amyloid hypothesis is still being challenged due to the failure of several high-profile drug trials. A significant amount of evidence supports the potential role of p53 in AD pathogenesis, particularly due to the protein’s functional dysregulation and involvement in many AD pathways. PTMs are an important part of the normal regulation of p53 cellular function, and the presence of a conformational change in p53 induced by redox dysfunction in AD, leading to a loss of function in many cellular response pathways, is an indication of a central role in AD. Further studies on the conformational unfolding of p53 showed that the conformational change of the protein impacts on its role, directly increasing or decreasing the activation of specific pathways involved in AD. A recent study has also shown that p53 is expressed in the AD brain in a phosphorylated isoform in correlation with p-Tau (Farmer et al., 2020). This study supports the effect of PTMs on p53 conformation and its role in AD. Recent studies (Abate et al., 2021; Piccirella et al., 2021) also showed the expression of a conformational variant of p53 in the plasma samples of asymptomatic and AD prodromal individuals confirming the role of the conformational variant of p53 in AD.

Further studies will be necessary to further elucidate the mechanism of actions for the specific PTMs of p53 and their involvement in AD pathways.

## Author Contributions

JC, SP, and PK defined the review focus. JC and SP wrote the manuscript. GA, PK, DU, and RK provided approval for publication of the content. All authors contributed to manuscript revision, read, and approved the submitted version.

## Conflict of Interest

JC, PK, and SP were employed by Diadem srl. The remaining authors declare that the research was conducted in the absence of any commercial or financial relationships that could be construed as a potential conflict of interest.

## Publisher’s Note

All claims expressed in this article are solely those of the authors and do not necessarily represent those of their affiliated organizations, or those of the publisher, the editors and the reviewers. Any product that may be evaluated in this article, or claim that may be made by its manufacturer, is not guaranteed or endorsed by the publisher.
